# Prenuclear L^∗^+H Activates Alternatives for the Accented Word

**DOI:** 10.3389/fpsyg.2019.01993

**Published:** 2019-09-24

**Authors:** Bettina Braun, María Biezma

**Affiliations:** ^1^Department of Linguistics, University of Konstanz, Konstanz, Germany; ^2^Spanish and Portuguese Department, University of Massachusetts Amherst, Amherst, MA, United States

**Keywords:** intonation, processing, contrastive topics, alternative sets, German

## Abstract

Previous processing studies have shown that constituents that are prosodically marked as focus lead to an activation of alternatives. We investigate the processing of constituents that are prosodically marked as *contrastive topics*. In German, contrastive topics are prosodically realized by prenuclear L^∗^+H accents. Our study tests (a) whether prenuclear accents (as opposed to nuclear accents) are able to activate contrastive alternatives, (b) whether they do this in the same way as constituents prosodically marked as focus with nuclear accents do, which is important for semantic modeling, and (c) whether the activation of alternatives is caused by pitch accent type (prenuclear L^∗^+H as contrastive accent vs. prenuclear L+H^∗^ as non-contrastive accent) or by differences in F0-excursion (related to prominence). We conducted two visual-world eye-tracking studies, in which German listeners heard declarative utterances (e.g., *The swimmer wanted to put on flappers*) and watched displays that depicted four printed words: one that was a contrastive alternative to the subject noun (e.g., *diver*), one that was non-contrastively related to it (e.g., *sports*), the object (e.g., *flappers*), which had to be clicked, and an unrelated distractor. Experiment 1 presented participants with two naturally produced intonation conditions, a broad focus control condition with a prenuclear L+H^∗^ accent on the subject and a contrastive topic condition with a prenuclear L^∗^+H accent. The results showed that participants fixated more on the contrastive alternative when the subject was produced with an L^∗^+H accent, with the same effect size and timing as reported for focus constituents. Experiment 2 resynthesized the stimuli so that peak height and F0-excursion were the same across intonation conditions. The effect was the same, but the time course was slightly later. Our results suggest that prenuclear L^∗^+H immediately leads to the activation of alternatives during online processing, and that the F0-excursion of the accent lends little. The results are discussed with regard to the processing of contrastive focus accents and theories of contrastive topic.

## Introduction

In intonation languages, utterances may be produced with a series of pitch accents, i.e., tonal targets or movements that are associated with the stressed syllables of accented words, see Example (1) – stressed syllables are underlined.


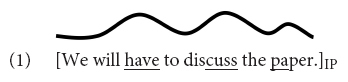


The utterance in (1) is produced as one intonation phrase (IP), i.e., without further phrasing. The last accent in an IP (or intermediate phrase, in languages that assume two layers of intonational phrases in the prosodic hierarchy, cf. Pierrehumbert, 1980; Grice et al., 2005) is called the nuclear accent. As detailed below, the nuclear accent has received particular attention in the prosodic, semantic, and processing literature. Particularly relevant for this paper is the finding that nuclear accents with certain pitch accents make alternatives more accessible (Weber et al., 2006; Ito and Speer, 2008; Braun and Tagliapietra, 2010; Husband and Ferreira, 2012, 2016; Gotzner et al., 2013; Gotzner, 2014). That is, listeners think of concepts that are contrastively related to the word bearing certain types of nuclear accent (see below), which results in priming effects and more fixations to contrastively related words or objects. Within the semantics/pragmatics literature, it is argued that nuclear accents determine the information structural category of a constituent as focus (shorthand “F”), where a focus constituent is a constituent that evokes alternatives relevant for interpretation (Rooth, 1985, 1992; Krifka, 2008).^1^ In this study, we deal with prenuclear accents that signal a contrastive topic interpretation as in the German example in (2) and test whether these accents activate alternatives and if so, whether they do so in the same way as nuclear accents do [unless otherwise indicated, the label *contrastive topic* and the shorthand “CT” is used to refer, descriptively, to constituents with a special prosody that forces a particular interpretation, spelled out by the optional follow up between parenthesis in (2) and whose prosodic features are not of our concern here].


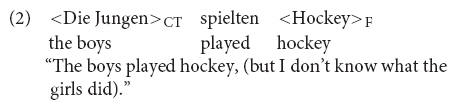


In short (see below for a more detailed discussion on the semantics and interpretation of contrastive topic utterances within the semantics and pragmatics literature), this special prosody (CT-prosody) indicates that the speaker decided to first say something only about a subset of the salient domain, e.g., only about *the boys* in (2), while there are other (contrastive) entities that s/he is not saying anything about.^2^ One question this paper tries to shed light on is the status of CT within information structure, i.e., whether this prosody identifies constituents as belonging to a basic notion of information structure (CTs would be then taken to encode a different information category from, e.g., focus), whether it is related to focus, or whether there is no need of an additional information category and focus can also cover these cases. This links the question of CTs as a (possible) notion of information structure with the question about what prosodic cues are used to activate alternatives, in terms of pitch accent type and phonetic realization.

In the remainder of the introduction, we first review the current state-of-the-art on the processing of nuclear vs. prenuclear accents (section “Nuclear vs. Prenuclear Accents”). We then turn to the concept of contrastive topics (section “Theories of Contrastive Topics”); they are interesting because they can be realized with a *prenuclear* accent in German, L^∗^+H, and because contrastive topics are claimed to trigger contrastive alternatives as well. In section “Intonational Realization of Contrastive Topics,” the prosodic realization of contrastive topics is reviewed, first for English, then for the target language German. It is shown that the contrast between contrastive and non-contrastive topics in German is realized on a continuum between L^∗^+H and L+H^∗^, with more acustically salient prosodic characteristics in contrastive than non-contrastive contexts, but that German listeners prefer prenuclear L^∗^+H in contexts that trigger a contrastive topic reading. In section “Outline and Hypotheses” we put forth the hypotheses regarding the activation of alternatives.

We then present two visual-world eye-tracking paradigm studies (Cooper, 1974; Eberhard et al., 1995; Tanenhaus et al., 1995), one with naturally produced contours – Experiment 1 – and one with resynthesized contours – Experiment 2 – to investigate four research questions, (a) whether subject constituents that are prosodically marked with prenuclear L^∗^+H lead to more fixations to a contrastive alternative than those marked with prenuclear L+H^∗^, (b) whether the fixation differences occur immediately while the constituent is processed and can hence be attributed to the pitch accent realization, (c) whether there is a difference in fixation pattern between contrastive topic and focus constituents, and (d) whether the activation of alternatives is caused more by pitch accent type or by its phonetic realization (in particular peak height and F0-excursion, which are related to perceived prominence). The answers to these questions will further our understanding on the role of prenuclear accents during speech comprehension, will allow us to contribute to the discussion regarding how to best formally model contrastive topics and overall to the discussion of the taxonomy of information structural categories, and to clarify the role of phonology and phonetic implementation in the activation of alternatives.

### Nuclear vs. Prenuclear Accents

The terms nuclear and prenuclear accent stem from the British School (e.g., Halliday, 1967a; Crystal, 1969; O’Connor and Arnold, 1973). In the nowadays dominant framework of autosegmental-metrical phonology (Pierrehumbert, 1980), all pitch accents have the same status. The difference between nuclear and prenuclear accent lies in their distribution in the utterance: nuclear accents form the head of the prosodic phrase and typically occur before a phrase break (intermediate phrase break in case there is one in the intonational phonology of the language, else intonation phase break), i.e., they are the last accent in the phrase. Prenuclear accents precede nuclear accents in the same phrase. In Example (3), if produced as a single phrase, there are hence two prenuclear accents (H^∗^ and L+H^∗^) and one nuclear accent (L+H^∗^), followed by a low boundary tone (L−L%).


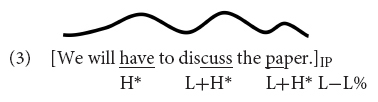


Nuclear accents have a number of interesting properties. First, they are more prominent to the listener than prenuclear accents, possibly owing to their special structural position. It has been shown that, if a prenuclear and a nuclear accent in the same phrase have the same F0-excursion, the nuclear accent sounds more prominent than the prenuclear one (e.g., Terken, 1991; Gussenhoven et al., 1997), see also Baumann and Winter (2018) for more recent evidence on German. Conversely, a nuclear accent needs less F0-excursion to be perceived as equally prominent as a prenuclear accent. Second, nuclear accents can signal focus and focal information is memorized better (Birch and Garnsey, 1995). Third, in terms of meaning contribution, the choice of nuclear accent type is claimed to signal differences in information status, i.e., whether a referent is new or accessible (e.g., Kohler, 1991; Baumann, 2006), focus location and domain (Eady and Cooper, 1986; Eady et al., 1986; Birch and Clifton, 1995; Baumann et al., 2006; Breen et al., 2010), illocution type (Braun et al., 2018b), as well as attitudinal information, such as sarcasm (e.g., Lommel and Michalsky, 2017).

The past approximately 20 years have accumulated knowledge on how nuclear pitch accents are processed online as the utterance unfolds over time (Dahan et al., 2002; Weber et al., 2006; Chen et al., 2007; Ito and Speer, 2008; Watson et al., 2008; Dennison and Schafter, 2010; Esteve-Gibert et al., 2016; Husband and Ferreira, 2016). In a frequently cited study, Dahan *et al*. (2002) investigated the effect of accentuation on reference resolution using the visual world eye tracking paradigm. Participants heard two instructions: In the first instruction, they were asked to move an object in a display (e.g., the *candle* in *Put the candle above the triangle*); according to a second instruction they had to move either the same object again (*candle*) or a lexical cohort competitor (*candy*). Object and competitor were either accented (nuclear H^∗^ or L+H^∗^, which was not controlled) or unaccented, resulting in four conditions. The results showed that before the cohort competitors were disambiguated segmentally, participants fixated the competitor *candy* more when the noun was accented, suggesting that listeners immediately exploited the relation between pitch accents and discourse structure for reference resolution. Notice that in Dahan *et al*. (2002) the experimental contrast was between a nuclear accent vs. no accent at all, which is a very prominent intonational contrast. Later studies have also shown that listeners are sensitive to smaller accentual contrasts, i.e., those between different *types* of nuclear accents (Chen et al., 2007; Watson et al., 2008). Moving from discourse effects to the immediate processing of pitch accents, Braun *et al*. (2018a) recently used the visual-world eye-tracking paradigm to test whether pitch accent type directly affects the fixation of contrastive alternatives, without an explicit context. In Experiment 1a of Braun *et al*. (2018a), German listeners heard declarative utterances (e.g., *The swimmer wanted to put on flappers*) and watched displays that depicted four printed words: one that was a contrastive alternative to the subject noun (e.g., *diver*), one that was non-contrastively related (e.g., *sports*), the object that had to be clicked (e.g., *flappers*), and an unrelated distractor. That experiment compared a nuclear L+H^∗^ accent on the subject [indicating that the subject was in focus, see Example (4)] to a prenuclear L+H^∗^ on the subject with a later nuclear accent on the object noun [indicating that the subject was part of a broad focus constituent, see Example (5)].


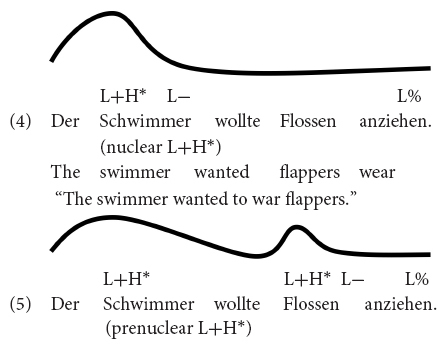


The results showed that participants directed more fixations to the contrastive alternative when the subject was realized with a nuclear L+H^∗^ accent [Example (4)] than when it was realized with prenuclear L+H^∗^ accent [Example (5)]. When the utterances were presented with a nuclear H+L^∗^ accent on the subject, an accent suitable to mark accessible information (Baumann and Grice, 2006), there was no difference in fixations compared to the prenuclear L+H^∗^. Also, there were no differences in fixations to the visually presented non-contrastive associate (e.g., *sports*). To account for the asymmetric fixation patterns for contrastive and non-contrastive associates, the authors argued against a priming account by which all kinds of related words are more strongly activated when the word is realized with a prominent nuclear accent (L+H^∗^). Instead, they concluded that the fixation data are better captured by the contrast in the semantic/pragmatic import of the two complex accents: the nuclear L+H^∗^ accent evokes contrastive alternatives while nuclear H+L^∗^ does not. Because there were differential results for the two nuclear accents L+H^∗^ and H+L^∗^, such that nuclear L+H^∗^ did and nuclear H+L^∗^ did not activate alternatives compared to prenuclear L+H^∗^, the authors argued that the fixation differences cannot be due to the status of the accents alone (nuclear vs. prenuclear), but to their interpretations.

Let us briefly discuss an alternative interpretation for the findings in Braun *et al*. (2018a), which will be addressed in more detail in this paper: the role of perceived prominence. According to e.g., Mixdorff and Widera (2001), accents with a higher peak are judged as more prominent in German (cf. Ladd and Morton, 1997 for English); this effect may not be due to peak height alone, but due to the increased F0-excursion of the tonal movement, as Gussenhoven (2002) pointed out: “[m]any perception experiments […] have shown that higher pitch peaks sound more prominent, everything else being equal. Interestingly, the effect is not simply due to peak height. Rather, it is an estimate of how wide the pitch excursion is, given some choice of pitch register, and the listener’s impression therefore results from an estimate of the pitch span in relation to some choice of pitch register” (Gussenhoven, 2002, p. 50). In the materials of Braun *et al*. (2018a), the nuclear accents L+H^∗^ and H+L^∗^ both had a higher peak and a larger F0-excursion than the prenuclear L+H^∗^ in the control condition: on average 9 semitones (st) for nuclear accents vs. 5st for the prenuclear accent. So pure peak height or F0-excursion cannot explain the fixation data in Braun *et al*. (2018a) either. However, we also know that pitch accent type matters for perceived prominence: Baumann and Röhr (2015) tested the prominence of a range of nuclear accent types that followed a prenuclear H^∗^ accent. Their findings showed that L+H^∗^ (with a F0-excursion of 5st) was judged most prominent, followed in prominence by L^∗^+H (also 5st) and H^∗^ (1.2st), all with ratings above 70 on a scale from 0 to 100 (from least to most prominent). H+L^∗^ (with an F0-excursion of 6st), the accent that did not result in fixation differences compared to prenuclear L+H^∗^ in Braun *et al*. (2018a), was judged to be less prominent (average prominence rating: 58), despite of its larger F0-excursion compared to nuclear L^∗^+H and L+H^∗^ accents. Prenuclear accents were not included in the prominence study by Baumann and Röhr (2015). In a more recent experiment, Baumann and Winter (2018) used the rapid prosody transcription task (Cole et al., 2010) and tested more varied sentence materials and also prenuclear accents. Their data showed that prenuclear accents were less often judged prominent than nuclear accents, but accent type and position (prenuclear/nuclear) were not orthogonally varied so it is not clear whether there is an interaction between the two factors. The perceived prominence of an accent may hence contribute to the activation of alternatives. This is in line with Calhoun (2009) who argued that the more phonetically prominent an accent the more likely a contrastive interpretation. We address the issue of prominence in the activation of alternatives in Experiment 2.

Prenuclear accents have generally been somewhat neglected in the semantic and processing literature, except for studies on their phonetic realization (e.g., Arvaniti et al., 1998; Atterer and Ladd, 2004). Semantically, prenuclear accents have been described as ornamental (Büring, 2007), serving a mostly rhythmic purpose (Calhoun, 2010; Chodroff and Cole, 2018). In a learning paradigm, Kapatsinski *et al*. (2017) showed that listeners focus more on the nuclear contour and largely ignore the prenuclear accents (cf. Roettger and Cole, 2018 for higher accuracy for whole contours and nuclear tunes compared to prenuclear accents in an artificial language paradigm). Prenuclear L^∗^+H accents may be an exception as this accent type is very prominent as a nuclear accent (Baumann and Röhr, 2015) and its inherent prominence may be used to trigger a CT-reading. This is the accent of interest in the present study.

### Theories of Contrastive Topics

There are different theories on how CTs are formalized. While (6) illustrates what is identified as *contrastive topic* constructions in the literature (which assumes a specific prosody that will be reviewed below for English and German), researchers differ on what they take contrastive topics to be and how they are interpreted. We overview the differences between the alternative approaches in (6a–c) below.


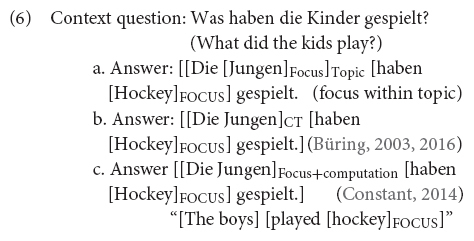


All researchers agree that the interpretation of the answers in (6), with the special prosodic features discussed below, can be paraphrased along the lines of “as for the boys, they played hockey” (following Jackendoff, 1972). In these utterances, *the boys* is what is called the contrastive topic constituent while *hockey* is the sentence’s (narrow) focus. However, researchers disagree on how we arrive at such an interpretation and on how many basic notions of information structure are necessary to model it (ultimately disagreeing on the taxonomy of information structural categories). These differences are what the contrast in (6a–c) tries to represent (we elaborate on these differences below). The results in this paper won’t allow us to discard any of the formal approaches to CT-constructions altogether, but they will allow us to critically evaluate different implementations of such approaches and narrow down the possibilities. On this respect, this paper tries to contribute to a discussion regarding how empirical investigations can inform formal and pragmatic modeling of CT-phenomena and narrow down the landscape. The hope is that future work will continue this discussion. We proceed below to evaluate the different formal approaches.

There are roughly two main camps in the formal semantics and pragmatics literature on contrastive topics (see also Constant, 2014 for an overview): those approaches that appeal to an independent notion of topic (syntactically, semantically or pragmatically defined) and that argue that a contrastive topic is a topic that contrasts with other topics (see Molnár, 1998; Vallduví and Vilkuna, 1998; Steedman, 2000; Krifka, 2008), and those who do not appeal to any independent notion of topic to understand contrastive topics (see, e.g., Gyuris, 2002, 2009; Büring, 2003, 2016; Tomioka, 2010a, b; Constant, 2014). In fact, a related question in the literature is whether CTs are basic notions of information structure or not. The discussion on CTs is part of a larger debate regarding the taxonomy of information structural categories. For some authors (see, e.g., Krifka, 2008) CTs are topic constituents containing focus (focus being a basic notion of information structure while the status of topic not being that clear). For others (see, e.g., Büring, 2003, 2016) CTs are a basic notion of information structure on their own. Finally, there are others (see, e.g., Tomioka, 2010b; Wagner, 2012; Constant, 2014) for whom CTs are just focus constituents. We provide a brief overview of these approaches and how they differ, and we hope that the sketches below can illuminate the discussion of the empirical results presented in this paper and how they contribute to the discussion of how to best formally model CTs. For the sake of concreteness, we focus below on Krifka (2008) as a representative of theories appealing to independent notions of topic to understand CTs, (6a). We dub this the *focus within topic* approach. We then sketch Büring (2003, 2016) and Constant (2014) as proposals in which understanding CTs does not require an additional notion of topic. These two proposals crucially differ on considerations regarding whether the taxonomy of information structural categories needs to contain both CT and focus (Büring, 2003, 2016), (6b), or whether the notion of focus is enough (Constant, 2014), (6c). We identify these last two approaches by the name of their respective proponents.

Let us start the discussion with the *focus within topic* approach as spelled out in Krifka (2008). Contrastive topics in Krifka (2008) are taken to be cases of *aboutness topics* containing an element marked as *focus*. In this approach to CTs we need both a notion of topic independently defined and a notion of focus. In Krifka’s view, the topic constituent is the constituent in the sentence identifying the entity or set of entities under which the information expressed should be stored in the common ground (understood in Stalnakerian terms as the information accepted by participants for the purpose of the conversation). This notion of topic is the notion of *aboutness topic* in Strawson (1964), Halliday (1967b), Reinhart (1981), Gundel (1988), Klein (2008) and goes together with a “structured” view of information update: when accepting the information communicated in an utterance we store it with respect to the topic entity, i.e., we identify the constituent in the utterance that is encoding what the utterance is about, the topic, and the constituent that is encoding what is being said about such entity, the comment, and store that for the given topic the comment has been predicated (this is, e.g., equivalent to the “link” in Vallduví and Engdahl, 1996). In the example in (6a), this would amount to identifying *the kids* as the topic and being able to organize information storage in such a way that we can store a bulk of information specifically about the kids. In particular, in (6a) we are asked to add the information that they played hockey. As for focus, in Krifka’s approach a focus element (where focus is a basic notion of information structure) is an element that evokes alternatives relevant for the interpretation [very much the proposal put forward in Rooth (1985), which is also the notion of focus in Büring (2003, 2016) and Constant (2014)]. CTs are then a combination of aboutness topic and focus. In the case of CTs the alternatives that are evoked are alternative topics, i.e., CTs are topics that contrast with other topics (Krifka, 2008, p. 45). Summing up, CT-interpretations are then arrived at by identifying a constituent as being the utterance’s aboutness topic and factoring in that it contains focus. This is what we will call the *focus within topic* account. In terms of processing, this view of contrastive topic is compatible with two formal implementations reflecting two processing procedures. One possibility is that conventional linguistic cues (in this case prosodic cues) could both identify a constituent as being the aboutness topic and as containing focus. In this approach the interpretation of the utterance as a contrastive topic would take place online. The other possible implementation involves arriving first at a complete syntactic analysis of the utterance (together with the information-structural analysis) to be able to identify the utterance’s aboutness topic and that the focus constituent is indeed within the topic. In this implementation contrastive topics are not processed online.

Let us see how this proposal differs from proposals in which the notion of CT does not depend on an independent notion of topic.^3^ Büring (2003, 2016) and Constant (2014) share important features regarding the interpretation of CTs. The interpretation of the sentences in (6b–c), assuming the special prosody discussed below, can be more precisely paraphrased as “as for the boys, they played hockey; the others, I’m not saying (because either I don’t know or because I don’t want to say).” Büring (and much subsequent work including Constant’s) follows the literature on formal discourse models (most importantly Roberts, 1996) and assume that utterances are embedded in a particular discourse structure, where discourse is a hierarchical order of moves organized around (implicit) questions that participants agree on addressing (discourse is a communal inquiry). The assumption in this approach is that “all that is given at the sentential level, conventionally, are certain sorts of presuppositions about the place and function of the utterance in the [intentional structure] of the discorse in which it occurs” (Roberts, 1996, p. 2). Following Rooth (1985), this literature takes focus to be one of the main conventional clues to link the utterance to discourse,^4^ since the focus structure of a particular utterance triggers the presupposition that there is a particular question open in the context that is being addressed (i.e., focus anaphora to a contextual question). That this is the case can be illustrated with question-answer pairs. The utterance in (7a) can be the answer to the spelled out question in (7), but (7b) can’t. The idea in focus theory is that even when the question is not spelled out, the focus structure allows us to identify what question the speaker is answering: (7a) and (7b) presuppose a different question in the context/discourse [the utterance in (7b) presuppose a question of the form *who drinks coffee?*].


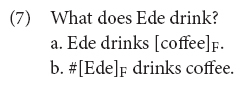


In this line of work, the utterance with CT-prosody presuppose a complex question: CT-utterances are analyzed as a partial answer to a (implicit) general complex inquiry of the form, e.g., *who did what?* The responses to the question in (6), assuming the specific prosody, signals that the speaker is resolving only a sub-issue (e.g., *what did the boys do?* in the running example) while s/he is leaving un-answered other contrastive sub-issues (e.g., a contrastive (implicit) question of the form *what did the girls do?*) that should be addressed to provide a complete answer to the complex question.^5^ In this way, the speaker is offering only a partial answer to the more general question. Considering that the question that speakers address in the discourse is the topic of conversation, Büring rightfully calls these utterances (as containing) *contrastive topics* (they address a (sub)-topic that contrasts with other topics). What differs between Büring’s and Constant’s work is how we arrive at this partial-answerhood interpretation. In Büring’s system (e.g., Büring, 1997, 2003, 2016), prosody reflects a specific marking in the syntax, CT-marking, see (6b), that comes with its own interpretational rules and lead to the right semantic interpretation (crucially, this marking is different from F(ocus)-marking in the Roothian sense and, hence, CT and focus are taken to be two independent notions of information structure). In Constant’s (2014) proposal (see also Tomioka, 2010b; Wagner, 2012), on the other hand, CT-phrases are no more than a F-marked phrase (in the Roothian sense) with special instructions regarding how the evoked alternatives enter into the semantic computation, see (6c).^6^ In Constant’s system, contrastive topic is not an independent category of information structure. Contrastive topic constituents are just focus constituents (i.e., F-marked constituents in the Roothian sense) plus some instructions regarding how the evoked focus alternatives are to be handled in the interpretation.^7^ In this sense, Constant’s proposal offers a simpler ontology of information structure categories.

What are the predictions made by these two theories? As said, Büring considers CTs as an information-structural category on their own. This alone may predict a different prosodic realization from F-phrases (the special prosody found in CTs would mark its status as a different information structural category). Notice, however, that in Büring’s theory the alternatives evoked by F-marked phrases and CT-marked phrases are different: syntactic F-marking evokes alternative propositions while syntactic CT-marking evokes alternative questions. In Constant’s system CTs are focus phrases. This approach hence makes the prediction that CT-phrases evoke alternatives in the same way as F-phrases do. Constant’s theory also makes predictions regarding the different prosodic realizations found in CT-phrases and F-phrases by virtue of their syntax. CT-phrases in Constant’s system are taken to be in the left periphery, either because they are moved there or because they are generated there, and it is this syntax that is responsible for the special prosody. How do we choose between the two systems? In what follows we sketch our reasoning in this paper.

The empirical investigation presented in this paper is related to how alternatives are activated in CT-constructions in contrast to what we find in narrow focus. That the alternatives that are evoked in CTs are different from those in narrow focus constructions (e.g., alternative propositions vs. alternative questions, as in Büring’s system) does not warrant a prediction that we should observe differences in the way alternatives are evoked/activated in contrastive topics constituents and focus constituents but, if we did observe such difference, we may consider it as partial support for contrastive topics being different from focus (against Constant’s proposal). At the same time, if there is no difference between how alternatives are evoked in contrastive topics constituents and focus constituents, we would lack support for a system that considers contrastive topics different from focus. That is, everything else being equal, if we are to choose between two systems, one simpler than the other, we need arguments to support that the more complex system is justified, e.g., in terms of processing. One way to do that is by showing that the way alternatives are evoked for contrastive topic and focus is different, explaining why we need two different information structural categories (cashed out formally in a different syntactic marking and interpretational mechanisms). If two models can derive the same results, in the absence of support for a more complex model we shall prefer the simpler approach.

Regarding how alternatives are evoked in CTs we investigate whether, as in the case of focus, alternatives are evoked online. Both (6b) and (6c) are compatible with alternatives being evoked online. However, for (6a) we saw that there are different possible implementations. The analysis is compatible with alternatives being evoked as soon as the accent is processed (online processing), but it is also compatible with late activation, once the listener has already assigned a syntactic analysis of the constituent as topic.

All proposals depicted in (6) predict that there is a difference between the answers in (6) and (8). Given the provided context-question, an exhaustive (neutral) answer^8^ is not expected to have the same prosodic marking as the CT-utterance in (6).


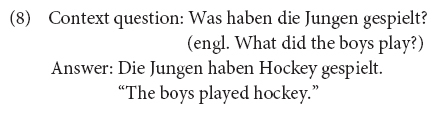


An important question addressed in this paper concerns the way we process utterances triggering CT-interpretations and whether this differs from the processing of focused constituents.

### Intonational Realization of Contrastive Topics

Since utterances with contrastive topic and focus constituents and broad focus utterances can have the same (surface) structure [see Examples (6) and (8)], when heard out of context, it is the intonational realization that distinguishes the interpretation of the grammatical subject as contrastive topic or focus or neither. Contrastive topics are often realized with different pitch accents from focal constituents. In English, Jackendoff (1972) described the prosodic realization of contrastive topics in English as B-accents (falling-rising contours) and foci as A-accents (falling contours), see Example (9). In the autosegmental-metrical framework, the B-accent contour is a complex phenomenon, represented as L+H^∗^ L−H% (authors also consider L^∗^+H as a possible complex accent for CT-phrases, see, e.g., Constant, 2014), while the A-accent contour is equivalent to H^∗^ L−L%, the prosodic realization of an exhaustive focus in English.


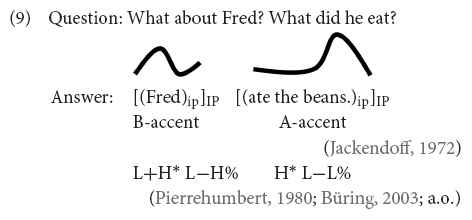


In German, however, contrastive topics are realized with a prenuclear rising L^∗^+H accent, while the (exhaustive) focus is realized as falling nuclear accent (Féry, 1993, p. 131). Unlike in English, there is typically no IP break between the contrastive topic and focus constituent (and hence there is no L−H% boundary tone). In German, the contrastive topic and the focus constituent are often produced in the same prosodic phrase. It is also often argued that the F0 contour between the rising accent on the contrastive topic and the fall on the focus remains high, resulting in the so-called hat pattern (originally described for Dutch by Cohen and ’t Hart, 1967)^9^. This realization is exemplified in Example (10), using the prosodic notation of the GToBI, German Tone and Break Indices, system (Baumann et al., 2001; Grice et al., 2005). German hence marks contrastive topics with a prenuclear accent. The prenuclear accent is prototypically an L^∗^+H, an accent that is judged as one of the two most prominent accents when placed in nuclear position (Baumann and Röhr, 2015). The nuclear accent on the focus constituent, H^∗^, is one that is not judged very prominent.


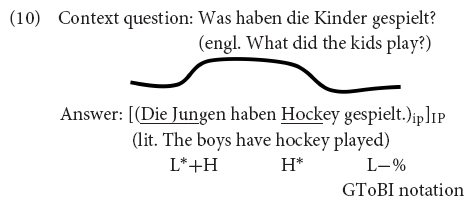


Experimental studies with identical sentences in different information structures showed that the prosodic difference between utterances identified as triggering a CT-reading [Example (10)] and those lacking this interpretation is not categorical (Braun, 2005, 2006, 2007). Instead, contrastive topics are typically realized with a later and higher peak and longer duration than the prenuclear rise in utterances without CT-interpretations. The hat pattern is not mandatory either. From the listeners’ perspective, while the prosodic contrast in the prenuclear accent in CT- and non-CT utterances is not necessarily categorical, prenuclear L^∗^+H is interpreted as contrastive topic, prenuclear L+H^∗^ is not. This was shown in a binary forced-choice context-matching experiment, in which participants received a written context (e.g., ‘*Jetzt geht es um einen Sohn und eine Tochter. Der Sohn beschäftigt sich mit Latein und…’* “The next story is about a son and a daughter. The son is occupying himself with Latin and…”) and heard a target sentence (*Die Tochter beschäftigt sich mit Mathe*. “The daughter is occupying herself with mathematics.”) in one of eight conditions, manipulating prenuclear accent type (L^∗^+H vs. L+H^∗^), nuclear accent type (H^∗^ vs. H+L^∗^) and the F0-transition between prenuclear and nuclear accent (high plateau vs. dip). The highest acceptance came from utterances with a prenuclear L^∗^+H accent and a nuclear H+L^∗^ accent, while the F0 transition between the two did not matter (81.6% for the high plateau, 89.3% for the dip). It is interesting to note that the preferred focus accent in CT-constructions in German is nuclear H+L^∗^, an accent type that is not judged particularly prominent in Baumann and Röhr (2015). In a context that did not trigger a CT-interpretation (CONTEXT: Die Tochter beschäftigt sich mit Mathe. “The daughter is occupying herself with mathematics.”, TARGET., weil sie morgen eine Klausur schreibt. “… because she will have a test tomorrow.”), participants gave highest agreement to contours with a prenuclear L+H^∗^ accent on the subject and a nuclear H^∗^ accent, irrespective of the F0 transition (69% for the high plateau, 68% for the dip). Given all these results, we will use a prenuclear L^∗^+H accent on the subject constituent and a nuclear H+L^∗^ accent as focus for the CT-condition in the experiments reported below. Since the F0 transition between the prenuclear and nuclear accent did not have an influence on perception, we stuck to one pattern, the hat pattern, which was more natural for the speaker. Regarding the *phonetic* implementation of prenuclear accents, offline acceptability studies have shown that participants find prenuclear rising accents with higher peaks more appropriate in contexts that triggered a CT-interpretation, accents with later but lower peaks were less acceptable but more appropriate than rises with earlier and lower peaks. In unmarked all-new contexts (Braun, 2004, 2005), there was no preference. Note that prenuclear L^∗^+H has also been reported as neutral prenuclear accent in Truckenbrodt (2002), who analyzed a not further specified sample of Southern German and Austrian speakers.

### Outline and Hypotheses

While the interpretation of the CT-constituent is often linked to contrast and some theories even link the CT-constituent directly to focus (see discussion above) this has not been supported by empirical findings in the literature yet. If CT-constituents were shown to activate alternatives, this would be the first demonstration that CT is processed like focus and that certain types of prenuclear accents (in addition to nuclear accents) have the potential to do so. Furthermore, depending on how this activation compares to the activation of alternatives found for utterances with narrow focus, the findings could provide empirical support to theories linking CT to focus in its treatment.

We use the visual-world eye-tracking paradigm with printed words (McQueen and Viebahn, 2007), which allows us to study the processing of contrastive alternatives without interference from visual relatedness (Huettig and McQueen, 2007). For the sake of comparability, we closely replicate Experiment 1a in Braun *et al*. (2018a), see examples (4) and (5). In Experiment 1 in this paper we compare two intonation conditions, naturally produced prenuclear L^∗^+H (contrastive topic, CT, condition) to naturally produced prenuclear L+H^∗^ (broad focus control condition). We measure participants’ fixations toward these referents while they process utterances in the two intonation conditions. A higher number of fixations to the contrastive associate in the contrastive topic compared to the control condition is interpreted as increased activation of the contrastive alternative in the contrastive topic condition. Note that the term “activation” is understood here as shorthand for “consider as lexical or conceptual alternatives,” In Experiment 2, we manipulate the intonation contours (PSOLA resynthesis) to reduce phonetic differences between contours.

Based on the semantic literature and the available processing data, we pose the following hypotheses on the activation of alternatives. The literature reviewed above results in a number of conflicting hypotheses on the role of prenuclear accents in processing (H1), on the comparison of contrastive topics accents and focus accents (H2), and on the role of F0-excursion of an accent for the activation of alternatives (H3). In what follows, we briefly lay out the possible hypotheses and advance some possible points of contention working against them.

H1. The available processing literature suggests that prenuclear accents are not processed as deeply (semantically) as nuclear accents. From that perspective, one would expect no differences in fixations between prenuclear L^∗^+H and prenuclear L+H^∗^. However, since prenuclear L^∗^+H has the potential to signal CT-constituents (among other things), we predict that prenuclear L^∗^+H leads to more fixations to the contrastive associate than prenuclear L+H^∗^ accents.
H2. Given that prenuclear L^∗^+H leads to a CT-reading, according to semantic/pragmatic proposals we predict that this accent has the same potential to activate contrastive alternatives than the nuclear L+H^∗^ focus accent of Experiment 1a in Braun *et al*. (2018a). If CT equals focus, we expect a similar effect size and a similar timing as for the focus data of Experiment 1a in Braun *et al*. (2018a).
H3. If a large F0-excursion is the decisive factor for the activation of alternatives, we predict that the fixation difference disappears when using resynthesized stimuli with the same F0-excursion of the rise for prenuclear L^∗^+H and L+H^∗^. These two accents did not differ in perceived prominence in Baumann and Röhr (2015) in nuclear position, where they had the same F0-excursion. If the interpretation of the accent type that is relevant, we hypothesize the same fixation differences between prenuclear L^∗^+H and prenuclear L+H^∗^ with resynthesized stimuli.

Hypotheses H1 and H2 are tested in Experiment 1, hypothesis H3 mainly in Experiment 2. Note that the experimental results with respect to H1 and H2 will allow us to discuss the different semantic/pragmatic formal theories in view of the psychological reality of contrastive topics.

## Experiment 1

### Methods

#### Participants

Forty native speakers of German between 19 and 33 years (average 25.7 years) participated for a small fee. Twenty-eight were female, 12 male. They were unaware of the purpose of the experiment and had not taken part in experiments involving similar materials. All participants reported to have normal hearing and had normal or corrected-to-normal vision. Written informed consent was obtained.

#### Materials

##### Sentences and visual displays

The experiment used the same sentence materials and displays as in Braun *et al*. (2018a). There were 24 experimental sentences and 24 filler sentences. All experimental sentences started with a subject-NP (see Table A1 in the Appendix), followed by a disyllabic auxiliary (*wollte “*wanted to”, *hatte “*had”, *konnte “*could”, and *sollte “*should”), an object noun and a non-finite verb (*Der Turner hatte Blasen bekommen* “The gymnast had gotten blisters”). Most of the subject-referents had penultimate stress and between two and four syllables. None of them had ultimate stress. The filler sentences were similar to the experimental sentences and also started with a definite subject-NP followed by a disyllabic auxiliary. However, they occassionally contained disyllabic verbs and temporal adverbials.

The words for the display in experimental trials had been selected as follows. For each of the subject nouns, there was one noun that was contrastively related and one that was non-contrastively related. The non-contrastive associate was collected in a free association task. Participants saw one noun at a time (e.g., gymnast), printed on screen, and had to type in the first word that came to their mind (e.g., sports). Due to this procedure of collecting highly active non-contrastive associates, these associates do not all have the same relation to the auditory target, i.e., some stand in a hyponym-hyperonym relation, others in a part-whole relation or refer to a typical instrument or location. While the hyponyms and hypernyms would qualify as replacements for the auditory target, the part-whole relations do not. It was not possible, however, to find enough non-contrastive associates with the same relation to the target. To collect the contrastive associate, participants saw a sentence fragment with a negated subject noun (e.g., “Not the gymnast had gotten blisters but the…”) and had to type in the most plausible continuation. For both the contrastive and the non-contrastive associates we chose the most frequent responses making sure that they differed from each other, were not onset competitors and had similar word lengths and lexical frequencies (factors that are known to affect fixation behavior, cf. Dahan et al., 2001; Kliegl et al., 2004). The average association strength, lexical frequency and number of characters of the selected contrastive and non-contrastive associates were matched, see Table 1. Each experimental trial showed the contrastive and non-contrastive associate, the grammatical object that had to be clicked as well as an unrelated distractor. The four words in any given experimental trial differed in onset letters.

**TABLE 1:** Average association strength, lexical frequency and number of characters (and standard deviations) of contrastive and non-contrastive associates to the subject nouns.

	**Contrastive associate**	**Non-contrastive associate**
Association strength (percentage)	30.3 (*SD* = 14.9)	27.9 (*SD* = 16.6)
Lexical frequency (occurrences per million)	1.5 (*SD* = 2.1)	4.6 (*SD* = 5.5)
Number of characters	6.8 (*SD* = 1.5)	5.9 (*SD* = 1.9)

In filler trials, the display showed the contrastive associate, the grammatical object that had to be clicked, a word that was non-contrastively related to the object and an unrelated distractor. In filler trials, the four words also differed in onset letters.

##### Recordings

**FIGURE 1:**
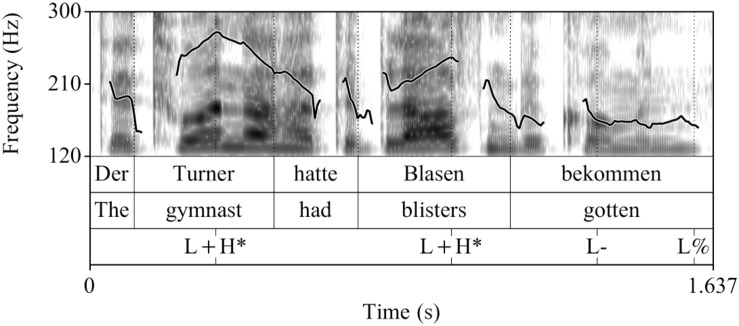
Example realization of a sentence recorded in the broad focus control condition (prenuclear L+H^∗^).

**FIGURE 2:**
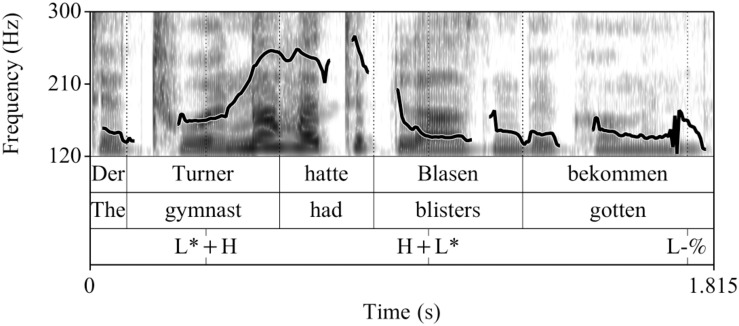
Example realization of a sentence recorded in the contrastive topic condition (prenuclear L^∗^+H).

The control condition (see Figure 1) and the fillers were the same as in Braun *et al*. (2018a). The experimental utterances (CT condition) were recorded anew, by the same female speaker of German under the same conditions (44.1 kHz, 16 Bit), see Figure 2. All sentences in the experiment were preceded by the prelude *Und ich habe gehört* “And I have heard,” to increase the preview time for the words in a natural way. This prelude was recorded once and spliced in front of all sentences with a pause of 1000 ms in-between.

Acoustically, prenuclear L^∗^+H (contrastive topics) differed from prenuclear L+H^∗^ in that they had a significantly later alignment of the L and H targets, a larger F0-excursion, and a longer duration of the stressed syllable, of the F0-rise and the entire subject-NP compared to prenuclear L+H^∗^. The mean values and standard deviations for each of these measurements in the two intonation conditions are listed in Table A2 in the Appendix. The sound files are availabe at Supplementary Data Sheets S1–S3.

#### Procedure

Intonation condition was manipulated as a within-subjects factor (but for every participant between-items), i.e., each participant saw all of the 24 experimental trials, but each target sentence was presented in only one of the two intonation conditions (totaling in 12 trials for each intonation condition). Across the experiment, the position of each of the different types of printed words was balanced (i.e., it occurred equally often in the upper left and right, lower left and right parts of the screen).

Two basic experimental lists were constructed, following a Latin Square Design. Each list further contained all the filler sentences. The two basic experimental lists were pseudo-randomized four times with the restriction of at most three experimental trials in a row (but at most two of the same intonation condition). After each block of five trials, an automatic drift correction was initiated. In total, we had eight experimental lists, to which participants were randomly assigned (five participants for each list).

Every trial started with a fixation cross which was shown until participants clicked on it. In all trials, the same token of the prelude (with a duration of 897 ms) was used. This was followed by a 1000 ms silence, after which the target utterance was auditorily presented. After participants had clicked on the respective object, there was a 1000 ms inter-trial interval. Eye-movement data (fixations, blinks, saccades) were recorded throughout the experiment.

The testing procedure was the same as in Braun *et al*. (2018a). Participants were tested individually in a sound attenuated room at the University of Konstanz. They were instructed in writing to listen to the utterances and to click on the object that is mentioned therein as quickly as possible. The instructions gave an example to make sure that participants knew what the object is.

Participants sat at a distance of approximately 70 cm from a 20 inch LCD screen, so that they could freely move the computer mouse. They rested their chin on the provided chin rest. Their dominant eye was calibrated with an SMI Eyelink 1000 system (pupil and corneal reflection at a sampling rate of 250 Hz). The same sampling rate was used during trials. The auditory stimuli were presented via headphones (Sennheiser PMX90) at a comfortable loudness.

### Results

**FIGURE 3:**
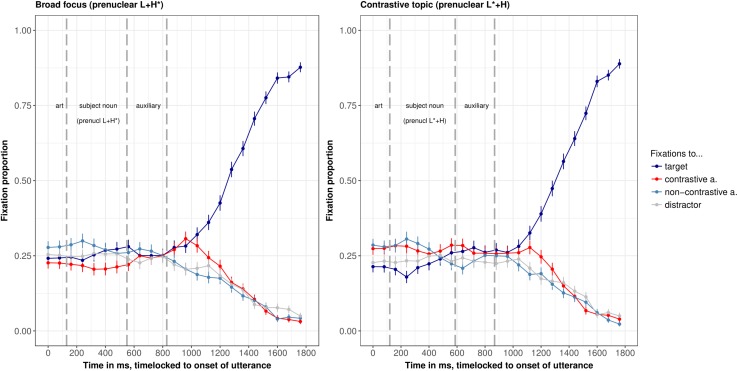
Grand averages of fixation proportions to the four words on screen, split by intonation condition (**left panel:** prenuclear L+H^∗^, **right panel:** prenuclear L^∗^+H), in 80 ms bins of Experiment 1. Whiskers show standard error. The line of interest is the red line, which shows fixations to the contrastive associate.

The eye-tracking data were processed as in Braun *et al*. (2018a). That is, the eye movement record was sampled in 4 ms steps and automatically parsed into saccades, fixations, and blinks by the EyeLink software (using normal saccade sensitivity). Only fixations were further processed. They were automatically coded as pertaining to a given word if they fell within a rectangle of 100 × 100 pixels, centered on the middle of that word. The grand average of evolution of fixations to the four words in the two intonation conditions is shown in Figure 3 (using the VWPre package in R, see Porretta et al., 2017). The gray vertical dashed lines indicate the segmental reference points, i.e., word boundaries from left to right. Note that it takes approximately 200 ms to launch a saccade (Fischer, 1992; Matin et al., 1993; Altmann and Kamide, 2004), which is also the delay in our studies: The fixations to the target (the grammatical object that had to be clicked, blue line in Figure 3) increased at approximately 1000 ms after utterance onset in the broad focus condition, i.e., approximately 200 ms after the onset of the grammatical object. The same delay of 200 ms is observed in the prenuclear L^∗^+H condition and is hence a good approximation for the time it took participants to launch saccades based on the auditory input. Hence, only after this time fixations can be interpreted as a response to the acoustic signal.

**FIGURE 4:**
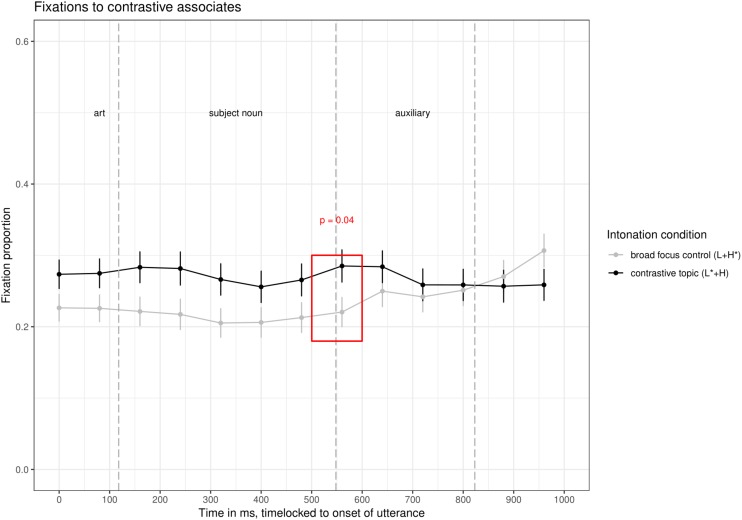
Comparison of fixations to the contrastive associate in the two intonation conditions of Experiment 1.

The interesting line for our research question is the red line in the time window from about 330 ms to 770 ms (i.e., 200 ms after the onset of the subject noun till 200 ms after its offset). This line shows fixations to the contrastive associate while participants were processing the subject noun. In Figure 4, the fixations to the contrastive associate in the two intonation conditions are compared directly.

For statistical analysis we analyzed participants’ fixations to the contrastive referent in consecutive 100 ms steps (cf. McQueen and Viebahn, 2007). We calculated the empirical logits of fixations to the contrastive associate in consecutive 100 ms windows starting from 100 ms after the onset of the utterance until 800 ms after its onset, dividing the fixations to that word by fixations that were directed elsewhere. A constant of 0.5 was added to both the denominator and the numerator (Barr et al., 2011). Empirical logits were analyzed using linear mixed effects regression models with *intonation condition* (prenuclear L^∗^+H vs. L+H^∗^) as fixed factor (dummy coded) and random intercepts for *participants* and *items* (Baayen, 2008; Baayen et al., 2008). The model further included random slopes for the two within-group factors when this improved the fit of the model, as determined by LogLikelihood comparisons, using the R-function anova(). *P*-values were calculated using the Satterthwaite approximation of degrees-of-freedom in the R-package lmerTest (Kuznetsova et al., 2016), which is based on lme4 (Bates et al., 2014).

In the time window 500–600 ms after the onset of the utterance, there were significantly more fixations to the contrastive associate in the contrastive topic condition (average logits = −1.7) than in the broad focus control condition (average logits = −2.3, ß = 0.56, *SE* = 0.19, *df* = 922, *t* = 2.9, *p* < 0.005), see Table 2 for *p*-values in all time windows. Note that there were no other significant differences in fixations to the contrastive associate in the entire time window shown in Figure 3. Given the time needed to plan a saccade, this difference is well within the time during which participants were processing the subject noun (170–270 ms after the onset of the subject noun, a period in time when all items are already unique when considering part-of-speech, grammatical gender, segments and stress, as indicated by a CELEX search). Note that fixations to the contrastive associate were numerically higher in the prenuclear L^∗^+H than in the prenuclear L+H^∗^ condition from the start of the utterance, but this difference was not significant. At the moment, we don’t have an explanation for this slight preference of the contrastive associate in the contrastive topic condition.

**TABLE 2:** Summary of *p*-values of comparisons of fixations to the contrastive associate (first row) and non-contrastive associate (second row) across intonation conditions in consecutive 100 ms analysis windows of Experiment 1.

	**100–200 ms**	**200–300 ms**	**300–400 ms**	**400–500 ms**	**500–600 ms**	**600–700 ms**	**700–800 ms**
Contrastive associate	*p* = 0.1	*p* = 0.1	*p* = 0.07	*p* = 0.1	***p* < 0.005**	*p* < 0.07	*p* = 0.3
Non-contr. associate	*p* = 0.9	*p* = 0.8	*p* = 0.1	*p* = 0.9	*p* = 0.4	*p* = 0.4	*p* = 0.2

Bold face indicates a significant difference at α = 0.05. The subject noun starts on average 130 ms after the onset of the sentence; it ends on average 580 ms after the onset of the sentence (averages over both intonation conditions).

In both intonation conditions, there were also many fixations to the non-contrastive associate, but these fixations to the non-contrastive associate were not affected by intonation condition (second row of Table 2). There were more fixations to the target (i.e., the grammatical object that had to be clicked) in the broad focus control condition than in the contrastive topic condition. This effect approached significance in the time windows from 200–500 ms after the onset of the sentence (see Table A3 in the Appendix). This is the opposite pattern as for the fixations to the contrastive associate, which suggests that target fixations are reduced in the contrastive topic condition because of increased fixations to the contrastive associate.

We then compared whether the effect of intonation condition was stronger here, in the prenuclear L^∗^+H condition than in the nuclear L+H^∗^ (contrastive focus) condition of Experiment 1a in Braun *et al*. (2018a). In that experiment, there was an effect of intonation condition in the same time window, but with a smaller magnitude (ß = 0.4 in Braun *et al*. (2018a) compared to ß = 0.56 in this experiment). To this end, we combined the data set and calculated the interaction between *experiment* and *condition* (contrastive topic/focus vs. broad focus control). The model showed no interaction between *experiment* and *condition* (*p* = 0.5); there was only a significant effect of *condition* in the combined data set, with more fixations to the contrastive alternative in the contrastive accents (nuclear L+H^∗^ and prenuclear L^∗^+H) than in the control condition (ß = 0.6, *SE* = 0.19, *df* = 1839.9, *t* = 2.9, *p* = 0.003). The lack of an interaction does not allow for strong conclusions. An additional Bayes Factor analysis indicated that the simpler model was more than 200 times more likely than the model with the interaction (Morey and Rouder, 2018). This suggests that the activation of contrastive alternatives is not different for nuclear L+H^∗^ accents and prenuclear L^∗^+H accents.

### Discussion

The eye-tracking data showed that participants fixated more on contrastive associates to the subject constituent when it was produced with a prenuclear L^∗^+H accent compared to a prenuclear L+H^∗^ accent. The difference was significant in the time window from 500–600 ms after the onset of the utterance, i.e., immediately while participants were processing the subject noun. We interpret these differences in fixations to the contrastive associate as evidence for an activation of alternatives upon hearing subjects with a prenuclear L^∗^+H accent as compared to prenuclear L+H^∗^. Given the lack of a difference for fixations to the non-contrastive associate, the data speak in favor of a model in which prenuclear L^∗^+H is a contrastive accent in the sense that it leads to an increased activation of contrastive alternatives. Note that this difference in fixations to the contrastive associate for prenuclear L^∗^+H (vs. prenuclear L+H^∗^) is the same as the difference in fixations reported for comparison of nuclear L+H^∗^ (contrastive focus vs. prenuclear L+H^∗^) reported in Experiment 1a in Braun *et al*. (2018a). It is of similar magnitude and occurs at the same time window, specifically between 500–600 ms after the onset of the utterance. The data hence suggest that nuclear L+H^∗^ and prenuclear L^∗^+H have the same potential to activate alternatives, vis-à-vis a non-contrastive prenuclear L+H^∗^ accent. This finding has interesting implications for the modeling of contrastive topics (see General Discussion).

We now focus on the time course of the effect of intonation condition to determine which part of the contour may have resulted in the activation of alternatives. We observe significant differences in fixations to the contrastive associate in the time window 500–600 ms after utterance onset. These fixations are triggered by acoustic information that occurred around 300–400 ms after utterance onset the latest (170–270 ms after the onset of the subject noun). This suggests that participants’ fixations are guided directly by the F0 information before and on the stressed syllable. Ritter and Grice (2015) already showed that German listeners are particularly sensitive to this “onglide” information, but only for nuclear accents. We add to this that prenuclear accents do not differ in this respect. Note that in this analysis window, only information on the pitch-level of the accented syllable is available (L^∗^ vs. H^∗^) and some information on the direction (rising or falling), but no information on the following pitch movement (dipping in broad focus, high plateau in contrastive topic condition). It hence seems that the pitch accent alone is sufficient to trigger the contrastive interpretation. This ties in with offline acceptability judgments, in which participants judged utterances with a combination of prenuclear L^∗^+H followed by a nuclear H+L^∗^ nuclear accent as more appropriate in a contrast that elicits a CT-interpretation, while the intervening pitch contour (the presence/absence of hat contour) had no effect (Braun and Asano, 2013). It is also consistent with findings on German that suggest that the onglide (the F0-information prior to the stressed syllable) is important for interpretation (Ritter and Grice, 2015).

## Experiment 2

Experiment 2 tested whether the differences in fixations to the contrastive alternatives are solely due to the differences in accent type (prenuclear L^∗^+H vs. L+H^∗^ here) or due to the differences in phonetic implementation of these accent types (in particular the peak height and the F0-excursion of the rise and the concomitant differences in perceived prominence). Since there are different opinions on whether prominence is related more to F0-excursion or the scaling of the tonal targets, we manipulated the F0-contour of both intonation conditions to make their F0-excursions (and the scaling of the low and high tonal targets of the accents) the same. Specifically, we (a) raised the low tonal target in the L^∗^+H condition, while keeping the high tonal target unaltered (making the CT accents less prominent under the view that L^∗^-accents are more prominent the lower the L-target and under the view that F0-excursion is related to perceived prominence) and (b) lowered the entire register of the L+H^∗^ condition, to have exactly the same F0-scaling for low and high tonal targets and a similar degree of unnaturalness induced by the resynthesis procedure.

### Methods

#### Participants

A different set of 40 speakers of German, recruited from the same subject pool, participated for a small fee. They were aged between 19 and 30 years (average 22.5 years, 32 female, 8 male). The participants were unaware of the purpose of the experiment and had not taken part in experiments involving similar materials. All participants reported to have normal hearing and normal or corrected-to-normal vision. Written informed consent was obtained.

#### Materials

The sentences and the visual displays were the same as in Experiment 1. All recordings were manipulated to achieve a similar F0-excursion for the contrastive topic and broad focus control stimuli and to achieve a matched sound quality. The recordings of the contrastive topic condition were first stylized [using the stylize pitch (2 semitones) function in praat, cf. Boersma and Weenink (1992-2011)]. Then, the low F0-values prior to the F0-rise were shifted up by 20 or 30 Hz, the choice depending on the naturalness of the resynthesis. Most utterances were shifted up by 30 Hz. The low F0-values after the nuclear accent were shifted up by the same amount. Furthermore, the F0-maximum was shifted up by 10 Hz for four recordings which had very low F0-maxima. The recordings of the control condition were also stylized and uniformly shifted down by 20 Hz, the fillers were stylized and shifted down by 10 Hz (a 20 Hz shift did not result in naturally sounding stimuli, so we sacrificed similarity of resynthesis procedure for naturalness in the case of fillers). This manipulation only changes the register. The acoustic realization of the resynthesized stimuli is shown in Table A4 in the Appendix. Crucially, the stimuli in the contrastive topic condition and the control condition did not differ in the F0-excursion of the pitch rise (*p* > 0.9), in the F0-value of the minimum before the rise (*p* > 0.3) and the F0-value of the maximum (*p* > 0.1).

**FIGURE 5:**
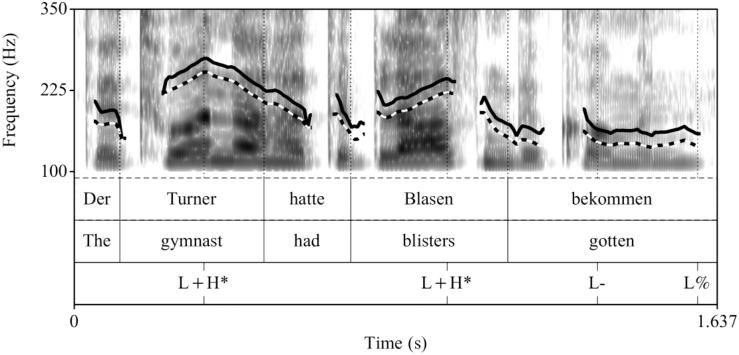
Comparison of F0-contours in the control condition (solid line: original contour of Experiment 1, dotted line: resynthesized contour of Experiment 2).

**FIGURE 6:**
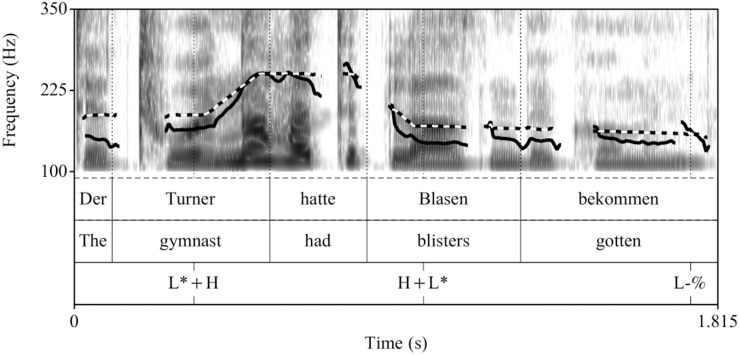
Comparison of F0-contours in the contrastive topic condition (solid line: original contour of Experiment 1, dotted line: resynthesized contour of Experiment 2).

Example comparisons between the resynthesized F0-contour across Experiments are shown in Figure 5 for the broad focus control condition and in Figure 6 for the contrastive topic condition.

#### Procedure

The experimental lists and the procedure were identical to Experiment 1.

### Results

**FIGURE 7:**
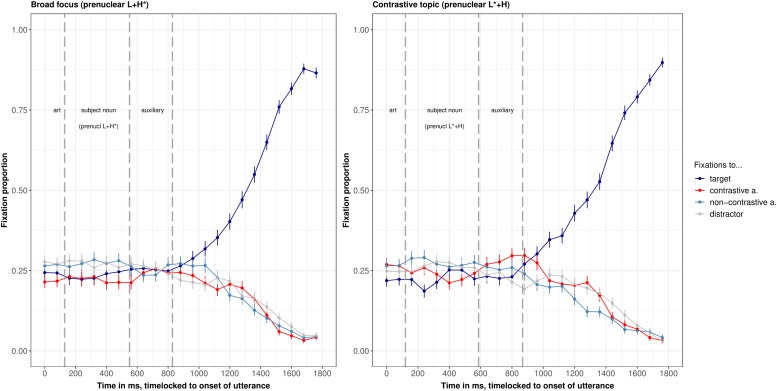
Grand averages of fixation proportions to the four words on screen, split by intonation condition (**left panel:** resynthesized prenuclear L+H^∗^, **right panel:** resynthesized prenuclear L^∗^+H), in 80 ms bins of Experiment 2.

**FIGURE 8:**
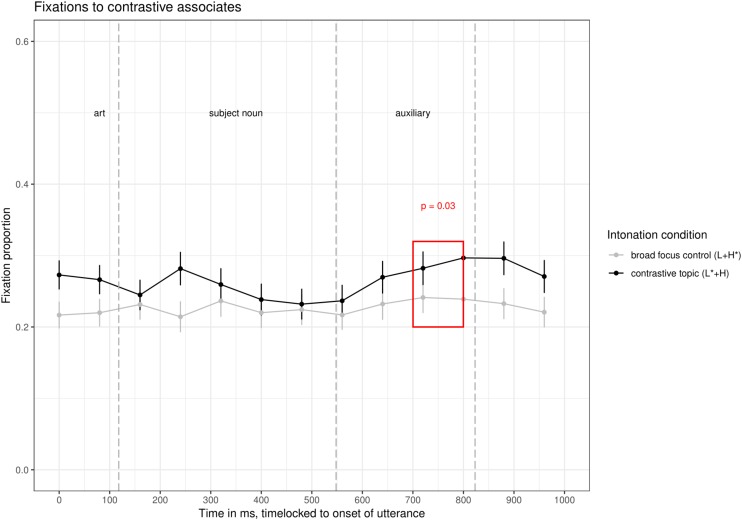
Comparison of fixations to the contrastive associate in the two intonation conditions of Experiment 2.

The evolution of fixations to the four words on screen over time is shown in Figure 7, the comparison of fixations to the contrastive alternative over time in Figure 8.

The results were analyzed in the same way as for Experiment 1. The analysis of fixations in subsequent 100 ms bins showed a significant effect of intonation condition in the time window from 100–200 ms and 700–800 ms (see Table 3, first row) after the onset of the utterance. In the 100–200 ms time window, participants’ fixations are not yet triggered by acoustic material from the stimulus, so it is difficult to understand the source of these differences. In the 700–800 ms time window, which clearly results from acoustic information in the subject noun, the average logits to the contrastive associate in the prenuclear L^∗^+H condition was −1.56, compared to −2.00 in the control condition (β = 0.43, *SE* = 0.2, *df* = 896, *t* = 2.1, *p* = 0.03). The time window of significant differences between prenuclear L^∗^+H and prenuclear L+H^∗^ is hence 200 ms later than in Experiment 1, while participants were starting to process the auxiliary following the subject. To test whether the differences in analysis windows between Experiment 1 and 2 are statistically significant, we pooled the data of both experiments and tested for an interaction between experiment and intonation condition. The interaction was not significant in any of the analysis windows (500–600 ms: *p* = 0.2, 600–700 ms: *p* = 0.8, 700–800 ms: *p* = 0.4). In all three analysis windows, there was only an effect of intonation condition (500–600 ms: *p* = 0.004, 600–700 ms: *p* = 0.02, 700–800 ms: *p* = 0.03).

**TABLE 3:** Results of statistical analysis of fixations in subsequent 100 ms time windows for Experiment 2.

	**100–200 ms**	**200–300 ms**	**300–400 ms**	**400–500 ms**	**500–600 ms**	**600–700 ms**	**700–800 ms**
Contrastive associate (Exp2)	***p* = 0.05**	*p* = 0.5	*p* = 0.7	*p* = 0.9	*p* = 0.2	*p* = 0.1	***p* = 0.03**
Contrastive associate (Exp1)	*p* = 0.1	*p* = 0.1	*p* = 0.07	*p* = 0.1	***p* < 0.005**	*p* < 0.07	*p* = 0.3

For comparison, the results of Experiment 1 are repeated in the second row. Bold face indicates a significant difference at alpha < 0.05.

Similar to Experiment 1 [and the Experiments in Braun *et al*. (2018a)], fixations to the non-contrastive associate did not differ across conditions (see Table A5 in the Appendix).

### Discussion

The results of Experiment 2 showed that pitch accent type (prenuclear L^∗^+H vs. prenuclear L+H^∗^) mattered for the interpretation and processing of subject constituents. As in Experiment 1, prenuclear L^∗^+H led to more fixations to the contrastive associate than prenuclear L+H^∗^, even though both contours were manipulated to have the same average F0-excursion in the rise. In combination with the data from Experiment 1 we can conclude that the exact peak height and F0-excursion had no influence on the presence of the effect. Statistically, the effect of intonation contour did not differ across experiments, but it is fair to acknowledge that the fixation differences reached significance later in Experiment 2 than in Experiment 1 (700–800 ms after the onset of the utterance in Experiment 2 compared to 500–600 ms in Experiment 1). Note that the time window at which the effect of intonation contour surfaced in Experiment 2 is one in which the processing of the noun is still taking place. Since the time it takes to plan a saccade is quite variable across listeners (Matin et al., 1993), it is also possible that some participants were already processing segmental information of the auxiliary and intonational information from the F0-transition (high vs. declining). Psychophonetically a high plateau following a rise has been shown to lead to the perception of peak delay (D’Imperio et al., 2010), which is a cue to contrastive topic interpretation, at least in offline studies (Braun, 2004).

We see two possible interpretations for why the effect of intonation occurs a bit later in Experiment 2. First, the resynthesized stimuli in Experiment 2 may take longer to process compared to the natural stimuli in Experiment 1. Previous research has already shown than a resynthesized and unfamiliar intonation contour slows down lexical access (Braun et al., 2011) and this may affect the activation of alternatives as well. This explanation predicts that any kind of unnaturalness in the stimuli leads to later effects, a prediction that can be tested in future experiments. Second, it is possible that – in the absence of a distinctive difference in the F0-excursion of the rise – the pitch accent contrast was blurred in the resynthesized stimuli and that listeners therefore used information on the F0-contour following the stressed syllable (high plateau in the case of prenuclear L^∗^+H and a declining pitch in the case of prenuclear L+H^∗^), a cue that by itself is not distinctive (Braun and Asano, 2013). The F0-information following the accented syllable disambiguates whether the L+H^∗^ accent is prenuclear or nuclear. In any case, the fixation data show that listeners activate contrastive alternatives for words produced with a prenuclear L^∗^+H accent even though its acoustic salience was reduced by reducing its F0-excursion (e.g., Mixdorff and Widera, 2001).

We now briefly turn to fixations to the non-contrastive associate. Once again, they did not differ in the two intonation contours, which lends further support to the assumption that only contrastive associates are affected by contrastive pitch accents. Experiment 2 has shown that prenuclear L^∗^+H is among the pitch accents that are processed contrastively, even when this accent had a reduced F0-excursion in the rise.

## General Discussion and Conclusion

Regarding hypothesis H1, which addressed the issue of whether *prenuclear* accents can in principle activate contrastive alternatives, the current fixation data showed that the prenuclear L^∗^+H accents in German do not differ from nuclear focus accents in this respect. Similar to nuclear focus accents, pitch accent type matters for whether or not contrastive alternatives are evoked. In both Experiments 1 and 2, listeners fixated more on contrastive alternatives to the subject noun (e.g., *diver* upon hearing *swimmer*) when it was produced with a prenuclear L^∗^+H accent (which may signal a contrastive topic interpretation) compared to a prenuclear L+H^∗^ accent (which is most compatible with a broad focus interpretation). Hence, claims in the literature that prenuclear accents are ornamental, mainly used for rhythmic purposes and remembered and processed poorly (Büring, 2007; Calhoun, 2010; Kapatsinski et al., 2017; Roettger and Cole, 2018) do not hold for all prenuclear accents alike. Clearly, in German, prenuclear L^∗^+H stands out in that respect. From a semantic/pragmatic perspective, this is not surprising, since theories of contrastive topic assume that CT-constituents (marked with prenuclear L^∗^+H in German) evoke alternatives. However, since many of those theories are on English, where the prosodic marking for a CT-interpretation includes a boundary tone (making the accent on the CT-constituent nuclear), it was unclear so far whether this formalization had to do with the fact that contrastive topics are realized with nuclear contours in English, which are known to activate alternatives, or whether it is the result of additional (e.g., syntactic) factors. Our data resolve this issue and indicate that prosodically marked CT-constituents do activate alternatives, even in a language in which CT-constituents are marked by prenuclear accents. In sum, the dichotomy between nuclear and prenuclear does not seem to be very informative for determining which accents are processed as contrastive and which are not.

Regarding H2, which addressed whether or not contrastive topics are processed in the same way as focus constituents with a nuclear L+H^∗^ accent on the subject (which were investigated in Braun et al., 2018a), the fixation data clearly show that there is no difference: both the effect size and the timing of the effects were similar. If anything, then the effect is even larger for contrastive topics than for focus constituents, but the cross-experiment comparison was not significant. To corroborate the proposal that contrastive topics behave like focus during online processing, it may be fruitful to investigate other properties that are attributed to the processing of focused constituents. For instance, focused constituents are processed faster than non-focused constituents (e.g., Cutler, 1976; Cutler and Foss, 1977; Cutler and Clifton, 1984), and remembered better (e.g., Gernsbacher and Jescheniak, 1995; Fraundorf et al., 2010). Similarly, while our data does not show a difference in how focus constituents with a nuclear L+H^∗^ accent on the subject and CT-constituents evoke alternatives, it would be very important to understand whether speakers treat CT-constituents differently from (standard) narrow focus constituents later on and, in this vein, whether CT-constituents differ from other constituents identified in the literature as more common (aboutness) topic constituents (marked syntactically or morphologically as such, e.g., by left dislocation in German or morphological marking in Japanese). Constituents more standardly understood as (aboutness) topic constituents are, for example, claimed to be better remembered than (standard) narrow focus constituents (see, e.g., Repp and Drenhaus, 2015).

These experimental data shed some light on theories of information structure and of contrastive topic. As outlined before, we take more fixations in the contrastive topic condition relative to the broad focus control condition as indication that the speaker is considering alternatives to the spelled-out element in generating the utterance’s interpretation (i.e., the element generating alternatives is). From this perspective, our fixation data show that the processing of CT-constituents is just like that of focus constituents. Given that in CT-constituents, as with focus constituents, alternatives are activated online as the utterance unfolds over time, and that L^∗^+H prenuclear accents indicate CT-interpretations (Braun, 2004, 2005; Braun and Asano, 2013), the results discard incarnations of the “focus within topic” proposals [see (6a) above, repeated here as (11a)] requiring that CT-interpretations are arrived at after full-syntactic processing and identification of the constituent as syntactic topic: given that constituents with L^∗^+H marking evoke alternatives online and are interpreted as contrastive topics, we can discard analyses in which we need to have a full syntactic analysis to then go back and interpret the L^∗^+H constituent as a contrastive topic.


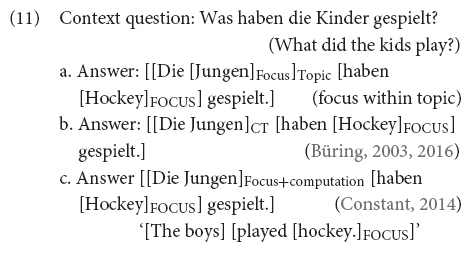


The results allow for incarnations of the focus within topic theory in which L^∗^+H both marks the constituent as focus and also identifies the constituent as a topic of some sort at the same time. This latter option would be equivalent, on this respect, to considering CT as a basic notion of information structure on its own (11b), and would also be compatible with a notion of CT as focus with special instructions regarding how to manipulate the evoked alternatives in the computation (11c). Regarding the contrast between predictions drawn from Büring’s proposal (11b) and those drawn from Constant’s proposal (11c), given that the experimental results show that the effect observed in processing CTs is similar to that in processing focus constituents, there is no empirical support from this data to maintain a more complex information structural taxonomy in which CT is different from focus. To be clear, the data does not discard (11b), but if F-marked elements are elements that evoke alternatives relevant for the interpretation and there is no difference between the activation of alternatives for the prenuclear L^∗^+H accent and the nuclear L+H^∗^ accent^10^, we do not find in these results support for a theory that considers two different notions of information structure, contrastive topic and focus, and a theory that subsumes the two under the same category is more appealing, i.e., (11c).

Our data also speak directly to hypothesis H3, which addressed the role of pitch accent type versus F0-excursion (which is related to intonational prominence) for the activation of alternatives. The fixations in Experiment 2, in which the stimuli were resynthesized so that the prenuclear pitch accents L^∗^+H and L+H^∗^ had the same F0-minimum, F0-maximum and F0-excursion, did not differ statistically from those of Experiment 1. This suggests that listeners did not directly react to the F0-excursion of the accents tested but processed the accent type (L^∗^+H vs. L+H^∗^). A closer inspection of the data shows that the effect occurred later in Experiment 2 with resynthesized stimuli than with the natural stimuli in Experiment 1. In section “Discussion” we discussed several options for the later occurrence of the effect in Experiment 2, such as general processing delays with resynthesized or unnatural stimuli as compared to natural stimuli, which are well documented in the literature (Braun et al., 2011). Due to the slightly different timing of the effect, participants had access to the information from the post-stressed syllable, which they lacked in Experiment 1. This may signal the listener whether the prenuclear L+H^∗^ accent in the control condition is in fact prenuclear or nuclear, a difference that mattered in Braun *et al*. (2018a). Although the transition/interpolation of F0 between the prenuclear and nuclear accent did not matter for participants when judging the appropriateness of the intonation contour in different contexts in an offline task, participants may be more affected in an online paradigm. Nevertheless, the available data pose a hen-and-egg problem: We do not know whether our F0-manipulation led to slower processing and hence to the availability of that information or whether the F0-manipulation jeopardized an important aspect of the pitch accent contrast (the onglide, cf. Ritter and Grice, 2015) so that participants had to use information on the F0-movement following the accented syllable. Overall, the data from Experiment 2 are compatible with an interpretation that pitch accent type (signaled by differences in tonal alignment) mattered more for the activation of alternatives than the peak height or F0-excursion of the pitch accents (and the prominence that goes along with these factors, cf. Mixdorff and Widera, 2001). Future studies are necessary to determine the relative strengths of individual prosodic cues that can signal a constituent as CT, also including non-tonal cues such as duration and intensity.

Taken together our findings show that prenuclear L^∗^+H on the sentence-initial subject, an accent that triggers a contrastive topic interpretation of the subject, leads to the activation of alternatives. This is the first study to show that a kind of prenuclear accent immediately evokes alternatives and that differences in accent type (alignment differences) matter for online processing irrespective of peak height or F0-scaling. Generally speaking, it is interesting to note that prenuclear L^∗^+H in Experiment 1 here, but not nuclear H+L^∗^ (Experiment 1b in Braun et al., 2018a), activated alternatives to the accented word, since both accent types share a common feature, that the stressed syllable is low-pitched. One explanation is that rising accents of this type are more prominent than falling accents (Baumann and Röhr, 2015; Baumann and Winter, 2018). This asymmetric pattern is mirrored by psychoacoustic studies on just noticeable differences (JNDs) for rising and falling contours (Jongman et al., 2017), who found that English listeners had lower JNDs for rising than falling contours, suggesting a heightened sensitivity for rising contours. This may also hold for German listeners. Yet, the nuclear H+L^∗^, which does not activate alternatives on its own, is the accent that is preferred after a contrastive topic (Braun and Asano, 2013). It is conceivable that the processing of contrastive topic constituents also affects the processing of the subsequent H+L^∗^-marked focus, such that listeners activate alternative to this focus constituent, too. It is an open issue why German, unlike English, identifies contrastive topic constituents with prenuclear and not with nuclear accents and why it uses an accent type (L^∗^+H) that, in nuclear position at least, is judged as less prominent than the prenuclear accent used in broad focus conditions (L+H^∗^). More work is necessary to unravel the effects of pitch and other suprasegmental cues to prominence (Baumann and Winter, 2018) and the role of prominence on the activation of alternatives.

## Ethics Statement

The studies involving human participants were reviewed and approved by the ERB of the University of Konstanz (30/2016). The patients/participants provided their written informed consent to participate in this study.

## Author Contributions

BB designed Experiment 1, statistically analyzed both experiments, and focussed on processing and prosody. MB and BB designed Experiment 2 together and worked on the Introduction and Discussion sections. MB focussed on semantics and pragmatics.

## Conflict of Interest Statement

The authors declare that the research was conducted in the absence of any commercial or financial relationships that could be construed as a potential conflict of interest.

## Footnotes

1 There are different notions of focus in the literature. The different notions of focus range from being the assertive part of an utterance (Lambrecht, 1994), the information that is new relative to the discourse (Firbas, 1975; Halliday, 1985; Vallduví and Engdahl, 1996), to being the constituent evoking alternatives (Rooth, 1992; Steedman, 2000; Krifka, 2008). In recent works, the information structure of utterances is typically established by showing congruence in specific question-answer pair contexts (e.g., Büring, 2009). These notions are not antithetic and can be subsumed under a common core, namely that a focus element is an element that evokes alternatives relevant for the interpretation (see Krifka, 2008 for discussion).

2 The optional follow up in (2) may also have the same prosodic features as the preceding conjunct, but it does not have to. If spelled out, given that the speaker is clearly dividing the set of entities between boys and girls and we already have the information about the boys, we do not need to use prosody to bring about the contrastive interpretation (e.g., “with respect to the girls specifically, I do not know what they did while about the boys I may know”). We will go in further detail below regarding the interpretation. What needs to be clear is that even though there are different ways to arrive at a contrastive-topic-like interpretation, the question we are focusing here is on how prosody enforces it and on how it can be formally modeled.

3 Büring (2003) takes the stronger position that a notion of *topic* is not necessary in general, while Constant (2014) argues that an independent notion of topic is not necessary to explain CTs but remains agnostic about how necessary it may be to account for other phenomena.

4 In the Roothian system constituents that generate alternatives relevant for the interpretation are F-marked syntactically; such marking is then reflected in the phonology, although how exactly this last step happens is open to debate. We have nothing to add to the theoretical discussion here. In Rooth’s system the focus structure of an utterance links the utterance to discourse by requesting that it be the answer to a question in discourse of a particular form; this is a form of *presupposition* and is cashed formally in Rooth’s work by the “∼” operator and its interaction with the focus meaning.

5 This more precise paraphrase is not applicable to *focus within topic* proposals of CTs. In Büring’s and Constant’s approach the formal system leads to the prediction that participants are (collectively) committed to also address the other sub-questions (i.e., to provide a complete answer for the more general complex question). In Krifka’s version of the *focus within topic* approach this is not encoded (although it can be derived as an inference).

6 Constant’s (2014) proposal falls into what he calls *configurational* accounts of contrastive topics, i.e., proposals that take a CT-phrase to be simply an F-marked constituent in a particular configuration. Other proposals of this sort include Tomioka (2010b) and Wagner (2012). The crucial difference between Constant’s (2014) proposal and other configurational accounts lies in the range of data the different proposals can explain within the characterization of CT (e.g., Wagner, 2012) argues that the configuration in which the constituent with a contrastive focus marking precedes the exhaustive focus is explained with different means from the configuration in which the contrastive topic constituent follows the exhaustive focus), as well as the predictions made regarding the phonology-syntax interface (i.e., Constant’s proposal can derive differences in the prosody of contrastive topic phrases vs. that of exhaustive focus, while those prosodic differences are not that easy to derive in other configurational proposals). The reader is referred to Constant (2014) for detailed discussion of the differences between different configurational proposals.

7 In Constant’s system the CT-phrase is either (covertly or overtly) moved to the left periphery or base generated there. The CT-phrase is an F-marked phrase that composes with the rest of the sentence via a CT-operator delivering the right interpretation within the (Roothian) focus dimension. This operator leaves the ordinary meaning intact. The desired interpretation of utterances with CT-marking is arrived at via old (Roothian) focus anaphora to a contextual question. In Büring’s system, we require a new dimension of meaning: besides the old focus meaning in Rooth’s system, Büring makes use of the CT-value, which requires its own compositional rules. Büring (2016) is explicit in that CT-interpretations are the result of a *conventional implicature* encoded in the CT-marking.

8 Exhaustivity can be derived either from the pragmatics of question-answer pairs or from a particular semantics for focus. Notice, however, that exhaustivity is not a feature of the Roothian focus-semantics.

9 In the German literature, this contour is known under the names *Hutkontur* “hat pattern” (Mehlhorn, 2001; Steube, 2001) “bridge contour” (Wunderlich, 1991; Büring, 1997), or *Wurzelkontur* “root contour” (Jacobs, 1982).

10 Note that the lack of a difference between the activation of alternatives for the prenuclear L^∗^+H accent and the nuclear L+H^∗^ accent is not a null effect in the classical sense of the term. The reason that there is no difference is that both prenuclear L^∗^+H and nuclear L+H^∗^ show equally strong fixation differences compared to the broad focus control condition, at the same time window.

## Supplementary Material

DATA SHEETS S1–S3 Sound files for Experiments 1 and 2.

## Appendix

**TABLE A1:** Subject noun (in German, broad phonetic transcription in IPA and English translation), together with contrastive and non-contrastive associate.

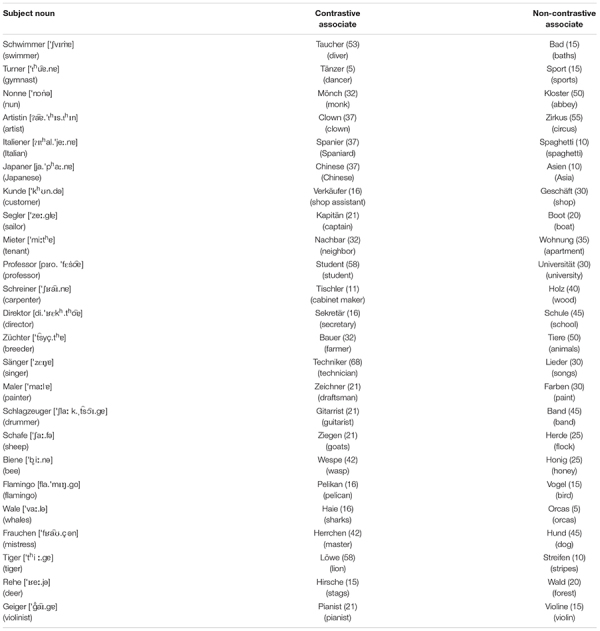

The number in brackets refers to the percentage of participants that named this associate in the web experiment (N = 19).

**TABLE A2:** Mean values and standard deviations in the two intonation conditions of Experiment 1.

	**Prenuclear L+H^∗^ in broad focus control condition (Experiment 1)**	**Prenuclear L^∗^+H in contrastive topic condition (Experiment 1)**
L-alignment with respect to start of stressed syllable in ms	−25.9 (48.5)	178.0 (57.9)
H-alignment with respect to end of stressed syllable in ms	−45.3 (33.2)	122.5 (50.1)
F0-excursion of the pitch rise in semitones	5.9 (1.1)	8.2 (1.3)
F0-minimum before the pitch rise in Hz	191.4 (8.1)	151.5 (8.5)
F0-maximum after the pitch rise in Hz	287.3 (13.8)	245.7 (8.6)
Duration of the stressed syllable in ms	247.5 (37.3)	272.6 (41.8)
Duration of the subject-NP in ms	421.0 (72.4)	457.8 (77.3)

**TABLE A3:** Summary of *p*-values of fixations to the target (Experiment 1).

	**100–200 ms**	**200–300 ms**	**300–400 ms**	**400–500 ms**	**500–600 ms**	**600–700 ms**	**700–800 ms**
Target (Exp 1)	*p* = 0.4	*p* = 0.05	*p* = 0.07	*p* = 0.05	*p* = 0.2	*p* = 0.6	*p* = 0.3

**TABLE A4:** Mean values and standard deviations in the two intonation conditions of Experiment 2.

	**Broad focus control condition (Experiment 2)**	**Contrastive topic condition (Experiment 2)**
F0-excursion of the pitch rise in semitones	5.7 (2.1)	5.8 (0.6)
F0-minimum before the pitch rise in Hz	184.4 (13.8)	179.3 (6.8)
F0-maximum after the pitch rise in Hz	254.6 (13.8)	249.9 (9.6)

**TABLE A5:** Summary of *p*-values of fixations to the non-contrastive associate (Experiment 2).

	**100–200 ms**	**200–300 ms**	**300–400 ms**	**400–500 ms**	**500–600 ms**	**600–700 ms**	**700–800 ms**
Non-contrastive associate (Exp2)	*p* = 0.2	*p* = 0.6	*p* = 0.8	*p* = 0.5	*p* = 0.7	*p* = 0.5	*p* = 0.4
